# Impact of UVR Exposure Pattern on Squamous Cell Carcinoma-A Dose–Delivery and Dose–Response Study in Pigmented Hairless Mice

**DOI:** 10.3390/ijms18122738

**Published:** 2017-12-16

**Authors:** Catharina M. Lerche, Katrine Togsverd-Bo, Peter A. Philipsen, Hans Christian Wulf

**Affiliations:** Department of Dermatology, Bispebjerg Hospital, University of Copenhagen, Bispebjerg Bakke 23, DK-2400 Copenhagen, Denmark; ktogsverdbo@dadlnet.dk (K.T.-B.); peter.alshede.philipsen@regionh.dk (P.A.P.); h.wulf@regionh.dk (H.C.W.)

**Keywords:** dose–delivery, dose–response, photocarcinogenesis, skin cancer, pigmentation, hairless mice, squamous cell carcinoma, SCC, UV radiation

## Abstract

Cumulative lifetime ultraviolet radiation (UVR) is an important factor in the development of squamous cell carcinoma. This study examines the impact of UVR exposure pattern on tumor development. Hairless C3.Cg/TifBomTac immunocompetent pigmented mice (*n* = 351) were irradiated with 12 standard erythema doses (SED)/week, given as 2 SED ×6, 3 SED ×4, 4 SED ×3, or 6 SED ×2 (dose–delivery study) or 0, 0.6, 1.2, 2, 3 or 4 SED ×3/week (dose–response study). All mice were irradiated until development of 3 tumors of 4 mm each. Pigmentation was measured once monthly. In the dose–delivery study, the median time until tumor development was independent of dose fractions. In the dose–response study, higher UVR doses resulted in faster tumor appearance. When the weekly UVR dose was decreased from 12 to 6 SED, the cumulative UVR dose needed for tumor development was reduced by 40%. In conclusion, delivery schedules of a fixed weekly UVR dose did not affect tumor development. When using different weekly UVR doses, longer time to tumor development was observed using lower UVR doses. Lower weekly UVR doses however resulted in lower cumulative UVR doses to induce tumors in hairless pigmented mice.

## 1. Introduction

Sunlight is the most important carcinogen for non-melanoma skin cancer, and especially squamous cell carcinomas (SCC), due to the cumulative effect of long-term ultraviolet radiation (UVR) in both humans and mice [1,2]. UVR exposure pattern differs among e.g., rural and urban children [3,4], occupations and outdoor sports activities [4]. Accordingly, it is often discussed whether the cumulative UVR represents the only health and safety parameter of importance for the development of keratinocyte cancers in humans, as well as whether the pattern of UVR exposure matters [5]. However, prospective investigations concerning UVR exposure pattern and skin cancer are, for obvious reasons, not possible to conduct in humans.

**Figure 1:**
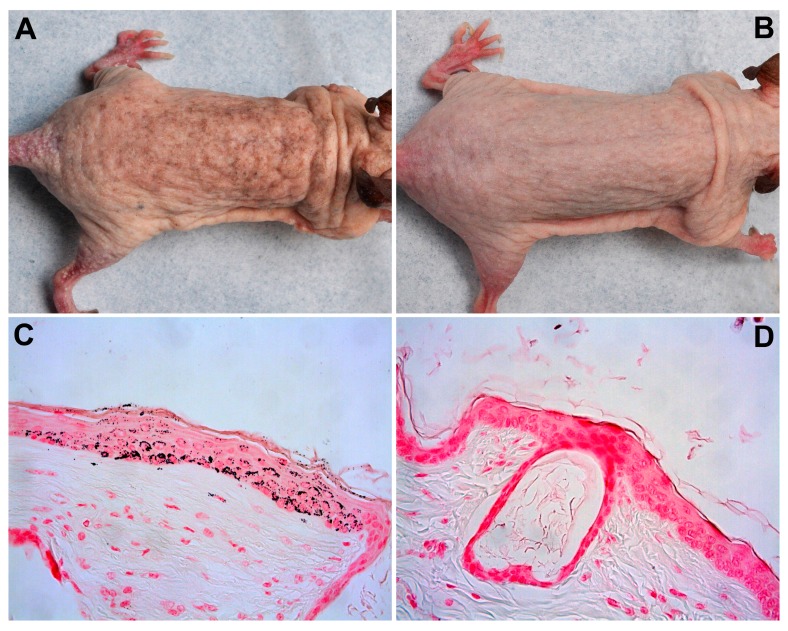
(**A**) Picture of a pigmented mouse; (**B**) Picture of a non-irradiated control mouse; (**C**) Melanin staining (Fontana-Masson ×40) of UV irradiated mouse skin [17]; (**D**) Melanin staining (Fontana-Masson ×40) of non-irradiated mouse skin with no visible melanin [17]. Figure 1C,D have been previously published [17], and are reproduced with permission from Catharina M. Lerche, Experimental Dermatology; published by Wiley and Sons, 2009.

Studies on UVR exposure patterns in humans and mice are reviewed by Martin et al., and show erythema-induction in human skin to be independent of exposure pattern, being only determined by the total administered radiant daily exposure; a concept termed reciprocity [6]. Studies of the importance of UVR exposure patterns on skin cancer in mice have mainly been conducted on hairless albino mice [7,8,9], but also, in some cases, on mice able to develop pigmentation [10,11]. In contrast to the erythema data on human skin, reciprocity has not been shown to hold in murine skin cancer studies [6]. For murine skin, therefore, carcinogenic effectiveness increases as irradiance is reduced or fractionated. Surprisingly, some studies show no or very little protection of induction of melanogenesis comparable to a sun protection factor of 1.5 [10]. Van Weelden et al. directly compared UVA carcinogenesis in SKH1 and SKH2 black and brown mice, finding median tumor latencies of 265, 267, and 375 days respectively. Thus, brown mice, but not black mice, were less susceptible than albino hairless mice [12]. The black pigmentation was noticeably patchy, which maybe correspond to lentigines in humans. This implies that protection provided by pigmentation is very variable over the skin. It is well-known that both mice and humans develop more pigmentation after higher UVR doses [13,14,15], and that pigmentation reduces photocarcinogenesis [16]. We have for many years used the C3.Cg hairless mouse, a strain which develops dose-dependent uniform pigmentation (Figure 1A,B). The pigmentation of this mouse is primarily situated in the epidermis (Figure 1C,D) [17], as is the case for humans [18]. However, dose–delivery and dose–response studies in this C3.Cg mouse strain have yet to be investigated.

The aim of this study was to investigate the relationship between (i) fractionated UVR doses, and (ii) different UVR doses on time to tumor development, and the cumulative dose at tumor appearance in the hairless C3.Cg mouse, a strain able to produce pigmentation. The present article consists of two parts: (A) a dose–delivery study, in which we investigated time to tumor development and the cumulative dose at tumor appearance using identical weekly UVR doses (12 standard erythema doses (SED)/week) given in different fractions; (B) a dose–response study, in which we investigated time to tumor development and the cumulative dose at tumor appearance using different weekly UVR doses in order to describe the dose–response curve for a wide, but still clinically relevant range of UVR doses (1.8–12 SED/week).

## 2. Results

**Figure 2:**
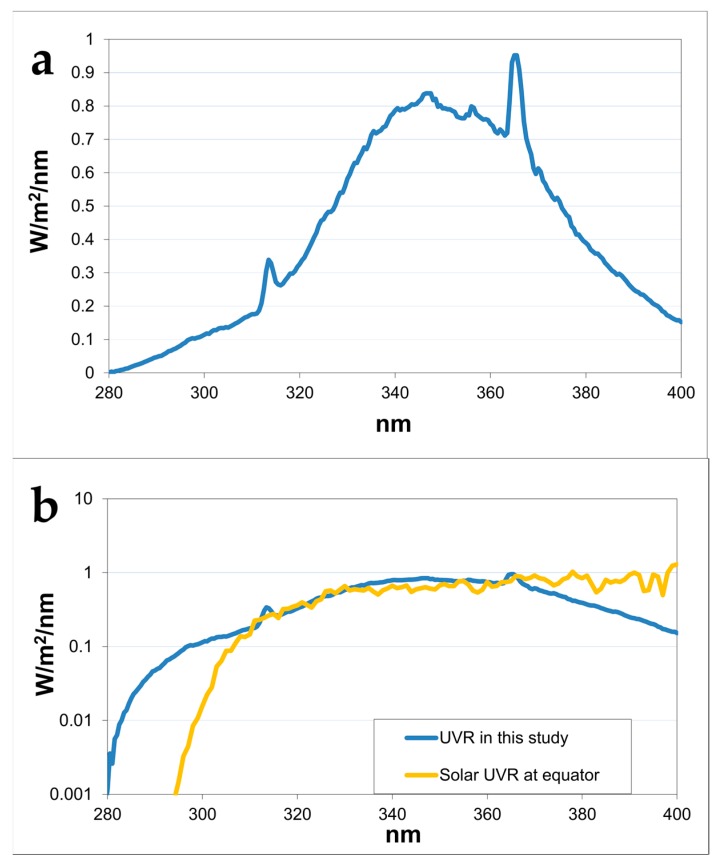
UVR spectra (**a**) Spectrum of the UVR used in this study, consisting of one Philips TL12 tube placed between five Bellarium-S SA-1-12 tubes; (**b**) Spectra from UVR used in this study (blue) and solar UVR at equator (orange) measured in the Spring, clear sky, solar height of 88° with an ozone layer of 250 DU and a UV-index of 11.6 (0.9 × 11.6 = 10.4 SED/h.

The irradiation regimens for the dose–delivery and dose–response study are shown in Table 1. The UVR spectrum used in the study is shown in Figure 2.

All UVR-irradiated mice except one developed tumors within the 500 day irradiation period. All histologically examined tumors histologically examined were SCCs. None of the mice in the non-irradiated Group 5 developed tumors. Table 2 shows the time until 50% of the mice in a given group had developed tumors, and the cumulative dose at the start of tumor development. There was no difference in weight development among the various groups of mice.

### 2.1. Dose–Delivery

#### 2.1.1. Time to Tumor and Cumulative Dose at Tumor Appearance

In the dose–delivery study, the median time until tumor development did not differ among Groups 1–4 (155, 176, 169, and 169 days, *p* ≥ 0.083) (Table 2 and Kaplan–Meier plot in Appendix). Mean cumulative dose at tumor development was likewise similar (Table 2).

#### 2.1.2. Pigmentation

**Figure 3:**
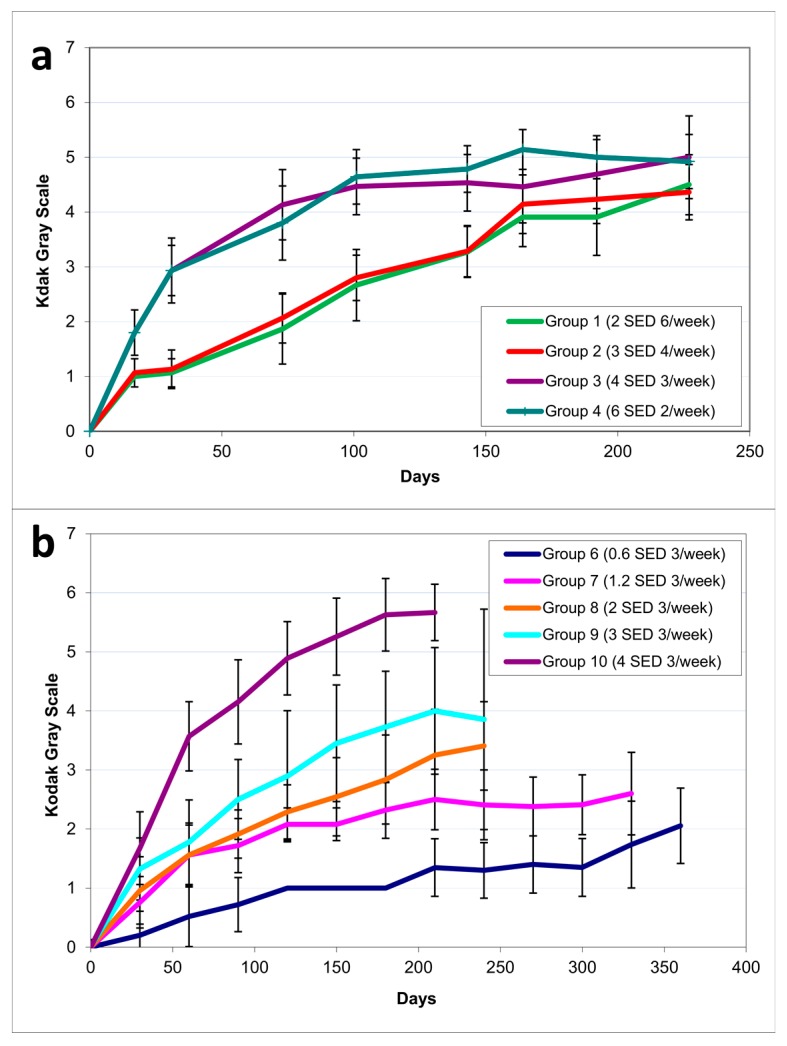
Mean development in pigmentation during (**a**) Dose–delivery study (Groups 1–4); (**b**) Dose–response study (Groups 6–10). Error bars indicate ± standard deviation (SD).

The development of pigmentation during the dose–delivery study is shown in Figure 3a. Groups 1–2 showed slower development in pigmentation compared to Groups 3–4, but reached almost the same level of pigmentation at the end of the study. The cumulative pigmentation at tumor development, henceforth called pigmentation, was calculated as the area under the curve (AUC) from the start of the experiment until the development of the first 1 mm tumor. (Table 2—Groups 1–4). There was no significant difference in pigmentation within Groups 1–2 and Groups 3–4, but there were significant differences between Group 1 and Groups 3–4, as well as between Group 2 and Groups 3–4 (*p* < 0.001).

### 2.2. Dose–Response

#### 2.2.1. Time to Tumor

**Figure 4:**
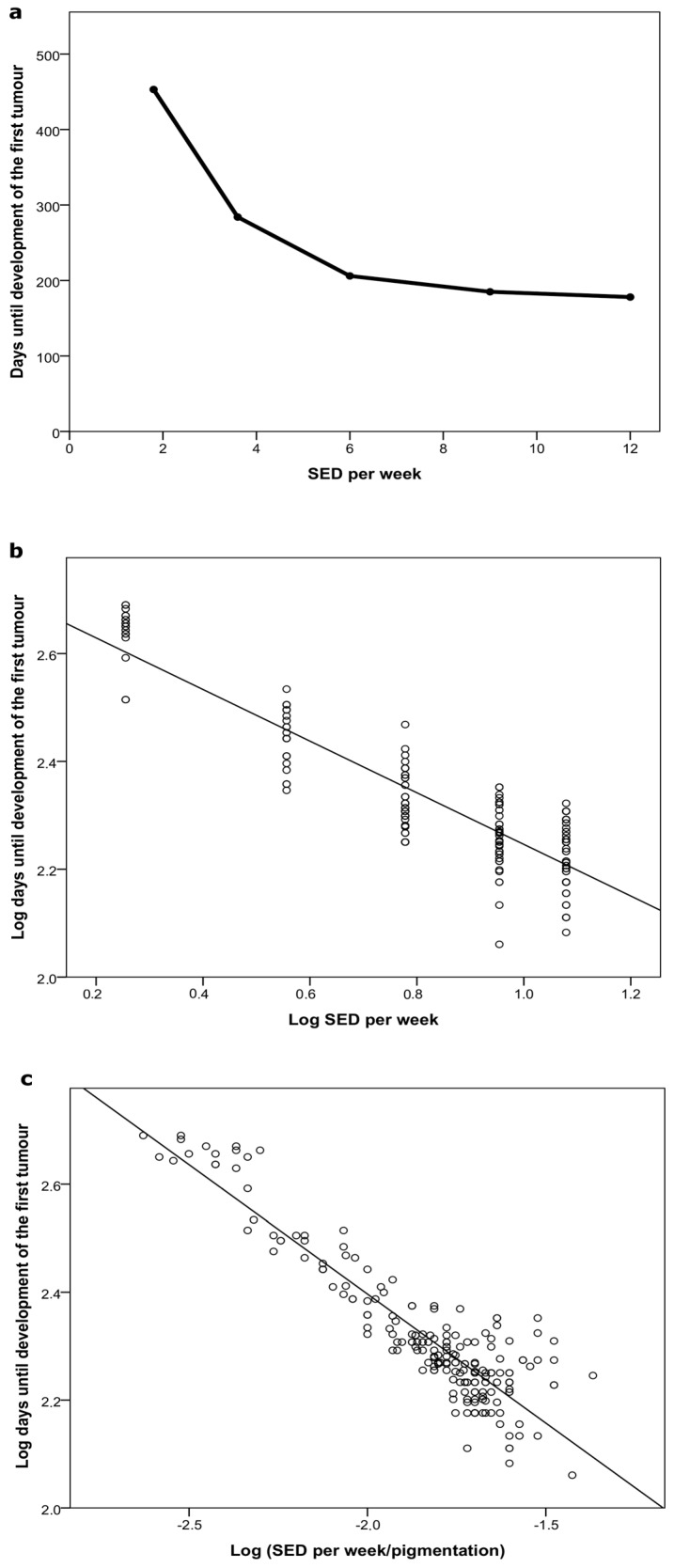
Dose–response curves (Groups 6–10) (**a**) Standard erythema doses (SED) per week as a function of median days until development of the first tumor; (**b**) Log_10_ SED per week as a function of log_10_ median days until development of the first tumor (y = −0.48X + 2.72, R^2^ = 0.802); (**c**) Log_10_ (SED per week/pigmentation) as a function of log median days until development of the first tumor (y = −0.48X + 1.44, R^2^ = 0.826).

In the dose–response study, there were significant differences in median time to tumor development among groups (Groups 6–10, *p* < 0.005) (Table 2, and a Kaplan–Meier plot in Appendix). The dose–response study shows a curved line between the dose and carcinogenic response when the mice were irradiated with different weekly UVR doses (Figure 4a). This relationship is linearized by taking the logarithmic function of the dose and time to tumor development (Figure 4b).

#### 2.2.2. Cumulative Dose at Tumor Appearance

**Figure 5:**
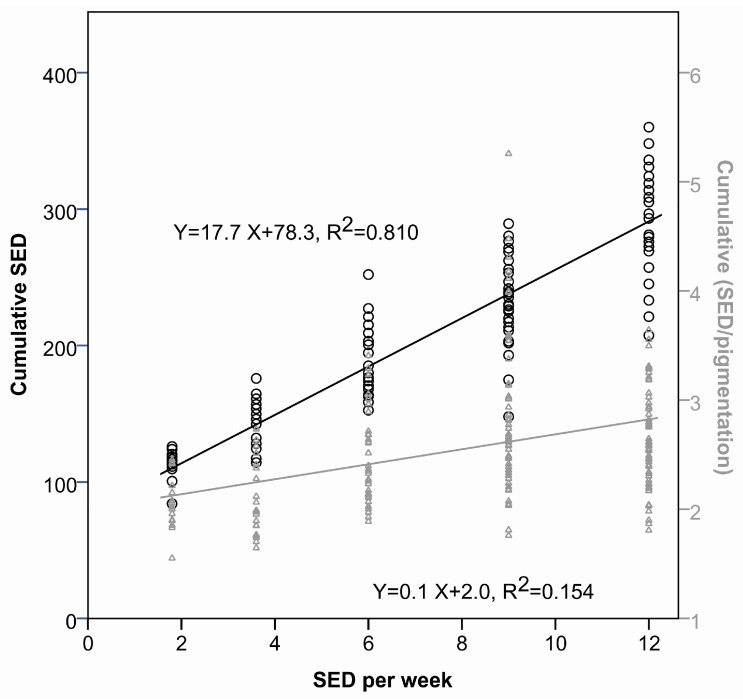
Linear relation between the cumulative dose at tumor development and the weekly irradiation dose (black). When mice receive fewer SED per week, they develop tumors after a longer time, regardless of the fact that the cumulative UVR dose are also lower. A linear relationship between the cumulative UVR dose corrected for pigmentation and the weekly UVR dose is shown in grey. This indicates that fewer SED per week still give tumors after lower cumulative UVR doses, but not as pronouncedly as before.

In the dose–response study, there were also significant differences in cumulative dose at tumor appearance among groups (Groups 6–10, *p* < 0.005) (Table 2). The cumulative dose when the first tumor appeared as a function of the weekly UVR dose are shown in Figure 5 (linear curve fit, Y = 17.7X + 78.3, R^2^ = 0.810). When the weekly UVR dose was decreased from 12 SED to 6 SED, the cumulative UVR dose needed for tumor development was reduced by approximately 40%.

#### 2.2.3. Pigmentation

The development in pigmentation during the dose–response study is shown in Figure 3b. As expected, higher UVR doses resulted in more rapid pigmentation development. There was no significant difference in cumulative pigmentation at tumor appearance in any of the groups except for Group 10, which was significantly more pigmented than the other groups (*p* < 0.0001).

Pigmentation reduces the UVR penetrating into the epidermis, and it will modify UVR dose for pigmented individuals. Thus, the linear dose–response curve in Figure 4b underwent adjustment for individual mouse pigmentation, and is depicted in Figure 4c. To investigate how important pigmentation and weekly UVR dose are for the cumulative dose at tumor development, we performed multiple linear regression analyses. These showed that the weekly UVR dose (R^2^ = 0.810) and pigmentation (R^2^ = 0.294) is of importance for the cumulative dose at the start of tumor development. Since pigmentation is a significant factor for the cumulative dose at tumor development, we also established the linear relation between the cumulative dose at tumor appearance and the weekly UVR dose, this time adjusting for the pigmentation (Figure 5, grey line, linear fit, Y = 0.1X + 2.0, R^2^ = 0.154). Findings demonstrated that the lower the weekly UV dose, the smaller the cumulative UV dose necessary for tumor development, though the relationship was less pronounced than before. When the weekly UVR dose was reduced from 12 to 6 SED, the cumulative UVR dose to tumor development decreased approximately 15%.

## 3. Discussion

Hairless mice constitute an internationally accepted model for the investigation of photocarcinogenesis and mice and humans exhibit similar responses to UVR [23,24,25,26]. This study investigated the carcinogenic effect of a few high UVR exposures compared with multiple smaller exposures (dose–delivery). Furthermore, the carcinogenic effect of very low, to higher but still tolerable doses of UVR, was also investigated (dose–response). Both studies were conducted in murine skin able to develop pigmentation.

### 3.1. Dose–Delivery

In the dose–delivery part of the study, we compared the effect of the same weekly UVR dose given as few high exposures, or as multiple smaller exposures, on photocarcinogenesis and pigmentation. We discovered that regardless of exposure pattern, equivalent weekly doses were equally carcinogenic, since we observed no difference in time to tumor development or cumulative dose by the time of first tumor appearance, despite a difference in pigmentation (Groups 1–4) (Figure 3a). Mice receiving a few high exposures of UVR had accelerated development in pigmentation (Groups 3–4) than mice receiving multiple smaller exposures (Groups 1–2). This finding is in accordance with previous reports describing longer time to development of pigmentation after suberythemal versus erythemal UVR doses [27]. However, the observed difference in pigmentation between Groups 1–2 and Groups 3–4 was apparently not enough to affect carcinogenesis. Previous studies in albino mice have shown that with a fixed weekly dose, fractionation increases the carcinogenic effect [9,28,29,30]. This could be due to an accumulation of DNA photodamage in the group with many suberythemal exposures, which has also been shown to occur in humans [31]. It could also be due to thickening of stratum corneum on exposure to UVR, which has a protective effect against further photodamage [32].

A weakness of our study setup is that we gave a weekly dose of 12 SED, which might be too high, according to the dose–response curve in Figure 4, even though 12 SED per week is less than 2 SED per day, and a dose that many people receive in their daily life [33]. We know from previous studies that these mice are able to develop tumors already after only 130 days, in combination with a chemical. Since it is thus possible to lower time to tumor appearance further, the model was not saturated in this study [22]. Nonetheless, it might have been better to administer a weekly dose of, for example, 6 SED in different fractioned doses, if the object was to observe a possible difference in tumor development. In conclusion, the dose–delivery study showed that exposure pattern for a fixed weekly UVR dose did not affect the tumor response. Therefore, we chose to irradiate the mice 3 times per week in the following dose–response study.

### 3.2. Dose–Response

Dose–response studies have mostly been conducted in hairless albino mice, and have shown a linear relationship between (a) time to tumor appearance and log dose [9,10], or (b) log time to tumor appearance and log dose [28]. Similarly, our dose–response study also shows a linear relation between log time to tumor and log weekly dose, R^2^ = 0.802 (Figure 4b). In Figure 4c, we have adjusted the dose for the individual pigmentation of each mouse in the study, resulting in an effective dose reaching each mouse. The R^2^-value increases to 0.826 in Figure 4c, compared with 0.802 in Figure 4b. We chose doses from 1.8 to 12 SED per week with a maximum of 4 SED per irradiation. One dose of 4 SED corresponds to the minimal erythema dose (MED) in mice and most Danes. The ambient UVR around noon on a Danish summer day (latitude 56° N) is 6 SED/h [33]. We did not use doses higher than 12 SED per week, since this would have increased the risk of skin damage and wounds in our mice. On the other hand, decreasing the dose below 1.8 SED per week would have resulted in a very long time until tumor development, and the risk of mice dying before this occurred increased.

Development of SCCs is believed to depend on UVR dose accumulated over a lifetime, and the relationship between dose and carcinogenic response is often thought to be linear, while exposure pattern is of less importance. In this murine model, SCC is dependent on the lifetime accumulated UVR. However, this study shows that mice receiving lower weekly UVR doses tolerate lower cumulative UVR doses before tumor development (R^2^ = 0.810, Figure 5). Thus, as UVR dose rises, the increase in carcinogenesis gets smaller, mostly because the level of pigmentation is especially induced by higher UVR doses. This is in agreement with previous publications stating that the tumor response of albino and pigmented SKH hairless mice is similar at low daily exposures, but that tumor development is less accelerated by increasing the daily exposure in the pigmented mice [8,10]. This could be due to the pigmented mice adapting far better to the higher daily exposures than albino mice [8]. The effect of pigmentation is confirmed by multiple regression analysis showing that both UVR doses and pigmentation are significant factors for cumulative dose at time of tumor development. If we adjust for pigmentation, thereby obtaining a more correct UVR dose (Figure 5—grey line), it still appears that the mice receiving lower weekly UVR doses develop tumors after lower cumulative UVR doses, but after longer time, and not as pronounced as before. When the weekly UVR dose is decreased from 12 to 6 SED, the cumulative UVR dose at tumor development is 40% lower, and when corrected for pigmentation, the cumulative UVR dose is 15% lower. From a previous publication on this mouse strain, it is known that the epidermis is about 2–3 times thicker after 6 SED/3 times per week for several months [17]. Thus, our findings could be explained by thickening of stratum corneum and epidermis by higher UVR doses [17,32].

A weakness of this study could be that groups were not treated simultaneously. Despite this, two groups receiving identical UVR doses (Groups 3 and 10) did not differ in time to tumor development (*p* = 0.455), cumulative UVR dose at tumor development (*p* = 0.211), or pigmentation (*p* = 0.143). Therefore, lack of concomitant treatment did not appear to affect results.

## 4. Materials and Methods

### 4.1. Animals

A total of 351 female C3.Cg/TifBomTac immunocompetent mice, aged 10–23 weeks at the beginning of the experiment, were obtained from Taconic (Ry, Denmark). The mice were sedated with 0.05 mL HypDorm (fentanyl citrate 0.158 mg/mL, fluanisone 5 mg/mL, midasolam 2.5 mg/mL), and tattooed with consecutive numbers on the abdomen. Each group (Table 1) was housed in separate boxes with free access to water and standard laboratory food. The mice were kept on a 12 h light/dark cycle in a 23–24 °C facility. This study was carried out in strict accordance with the recommendations in the Guide for the Care and Use of Laboratory Animals of The Danish Animal Experiments Inspectorate. The protocol was approved by the Danish Animal Experiments Inspectorate (permit number: 2004/561-823 approval date: 24 February 2004 ) and our Institutional Animal Care and Use Committee.

### 4.2. Light Sources

The UVR doses were expressed in standard erythema doses (SED) [34]. Irradiation regimens for the dose–delivery and dose–response studies are shown in Table 1. The UVR source consisted of one Philips TL12 tube (Philips, Eindhoven, The Netherlands) placed between five Bellarium-S SA-1-12 tubes (Wolff System, Atlanta, GA, USA) emitting 10.7% in the UVB range (erythema–weighted 97.4%) (Figure 2). The UVR emission spectra were measured with a spectroradiometer (Solatell Sola-Hazard 4D Controls Ltd., Cornwall, UK). Mice were irradiated through the wire lids on the tops of the cages, with a maximum of 25 mice per cage. UVR doses were given at fixed times.

### 4.3. Study Design

Mice were divided into groups (Table 1) and examined weekly for the presence of tumors. The size of each tumor was determined using calipers, and along with location, was recorded. For all experiments, the first four tumors on the back with a diameter of at least 1 mm were mapped separately for each animal. The duration of time until tumor development was defined as the number of days that passed until the first tumor of 1 mm in diameter appeared and later grew to 4 mm in diameter [35]. We did not record the total number of tumors, since tumors often grow into each other and form a carpet-like layer in which single tumors cannot be counted. The mice were sacrificed after development of one tumor with a diameter of 12 mm or 3 tumors with diameter of at least 4 mm, or after 500 days.

### 4.4. Pathology

The dorsal skin was removed and fixed in 4% buffered formaldehyde. A pathologist examined a minimum of two randomly selected mice from each UVR-irradiated group to confirm the diagnosis of squamous cell carcinoma.

### 4.5. Weight and Pigmentation

Weight and pigmentation were measured monthly. To quantify pigmentation, the mice were placed in a dark room under a bank of 6 Philips TL08 fluorescent UVA tubes (Philips, Eindhoven, The Netherlands) and the color of the pigmented skin was compared on a Kodak Gray Scale with 20 different, equally spaced shades (pigmentation degrees), from white to black [36].

### 4.6. Statistics

Time until tumor development was analyzed using a Kaplan–Meier plot (Appendix), and the groups were compared using log-rank tests (Mantel–Cox). Pigmentation was analyzed by calculating the area under the curve from study start until tumor development, because measurements are less accurate once tumors have appeared. Group comparisons of cumulative dose at tumor development and pigmentation at tumor development were performed using a one-way ANOVA test, followed by a post hoc test of individual comparisons using *t*-tests with Bonferroni correction.

Multiple regression analyses were performed to investigate whether pigmentation and irradiation dose were important factors for the cumulative dose at the start of tumor development. Non-linear regression (curve fit) was performed to assess the best relation between the cumulative dose and the irradiation dose. For all calculations, a *p*-value < 0.05 was considered statistically significant. All analyses were performed using IBM SPSS 22.0 for Windows (SPSS Inc., Chicago, IL, USA).

## 5. Conclusions

In conclusion, UVR exposure pattern was of no importance for tumor development using the same weekly UVR doses in the dose–delivery study. As expected, the dose–response study further showed that higher weekly UVR doses resulted in accelerated tumor development. Interestingly however, at the time of tumor appearance, cumulative UVR dose was smaller using low weekly UVR doses compared with the higher doses. At higher UVR doses, mice became more pigmented, providing increased protection against UVR’s carcinogenic effect. When we adjusted for pigmentation, lower UVR doses still led to lower cumulative UVR doses at tumor appearance, although the tendency was much less pronounced than before correction.

## Figures and Tables

**Table 1:** Treatment schedule. Standard erythema dose (SED), ultraviolet radiation (UVR).

Study	Group	Irradiation (UVR) Regimen Total SED per Week		*N* (Hairless Mice)	Previously Published (Reference)
Dose–delivery	1	2 SED 6×/week	12	15	-
	2	3 SED 4×/week	12	15	-
	3	4 SED 3×/week	12	15 *	-
	4	6 SED 2×/week	12	15	-
Dose–response	5	0 SED	0	50	[13,19]
	6	0.6 SED 3×/week	1.8	25	-
	7	1.2 SED 3×/week	3.6	25	-
	8	2 SED 3×/week	6	50	[19,20]
	9	3 SED 3×/week	9	75	[19,21,22]
	10	4 SED 3×/week	12	66 *	[2,19,20]

* Mice in group 3 and 10 are not identical, even though they have received the same dose regimen.

**Table 2:** Number of days until tumor development in 50% of the mice in each group. In the dose–delivery study, the median time until tumor development and the cumulative dose at tumor development did not differ among Groups 1–4, but there was a difference in pigmentation between Group 1 and Groups 3–4, as well as between Group 2 and Groups 3–4, (*p* < 0.001). In the dose–response study, the median time until tumor development and the cumulative UVR dose at tumor onset were significantly different in all the groups (Groups 6–10, *p* < 0.005). Pigmentation at the time of the first tumor was identical in all the groups, except for Group 5 (*p* < 0.0001). Standard erythema dose (SED). Interquartile range (Q_1_ = 75th percentile and Q_3_ = 25th percentile), arbitrary units (AU).

Study	Group	Irradiation Regimen	Median Days to 1st Tumor (Q_3_–Q_1_)	Mean Cumulative UVR Dose (SED) at Time Point of Tumor Development (SD)	Pigmentation (AU) at the Time Point of Tumor Development (SD)
**Dose–delivery**	1	2 SED 6×/week	155 (141–183)	273 (34.1)	316 (69)
	2	3 SED 4×/week	176 (155–183)	297 (41.2)	412 (69)
	3	4 SED 3×/week	169 (155–176)	279 (27.9)	586 (107)
	4	6 SED 2×/week	169 (169–190)	303 (52.1)	658 (153)
**Dose–response**	5	0 SED	No tumor	No tumor	No pigmentation
	6	0.6 SED 3×/week	453 (433–468)	113 (12.1)	489 (134)
	7	1.2 SED 3×/week	284 (257–313)	145 (17.4)	487 (122)
	8	2 SED 3×/week	206 (191–237)	182 (23.7)	428 (124)
	9	3 SED 3×/week	185 (171–199)	235 (27.7)	426 (125)
	10	4 SED 3×/week	178 (150–196)	293 (39.4)	648 (133)

**Figure A1:**
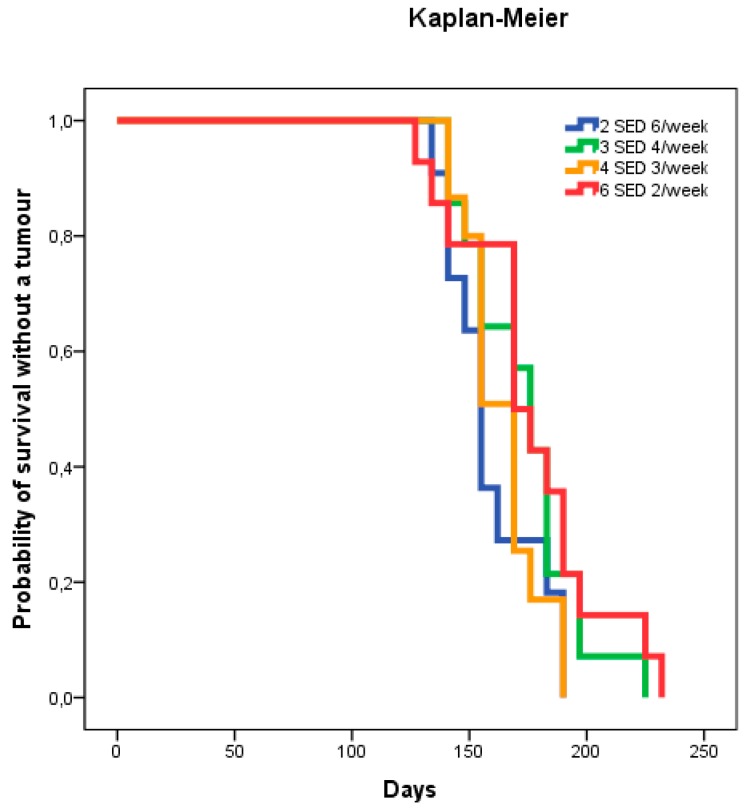
Dose–delivery study: Kaplan–Meier plot showing tumor-free survival (Groups 1–4). There are no differences in time until tumor development between the groups irradiated with 2 SED 6×/week, 3 SED 4×/week, 4 SED 3×/week, or 6 SED 2×/week (155, 176, 169, and 169 days).

**Figure A2:**
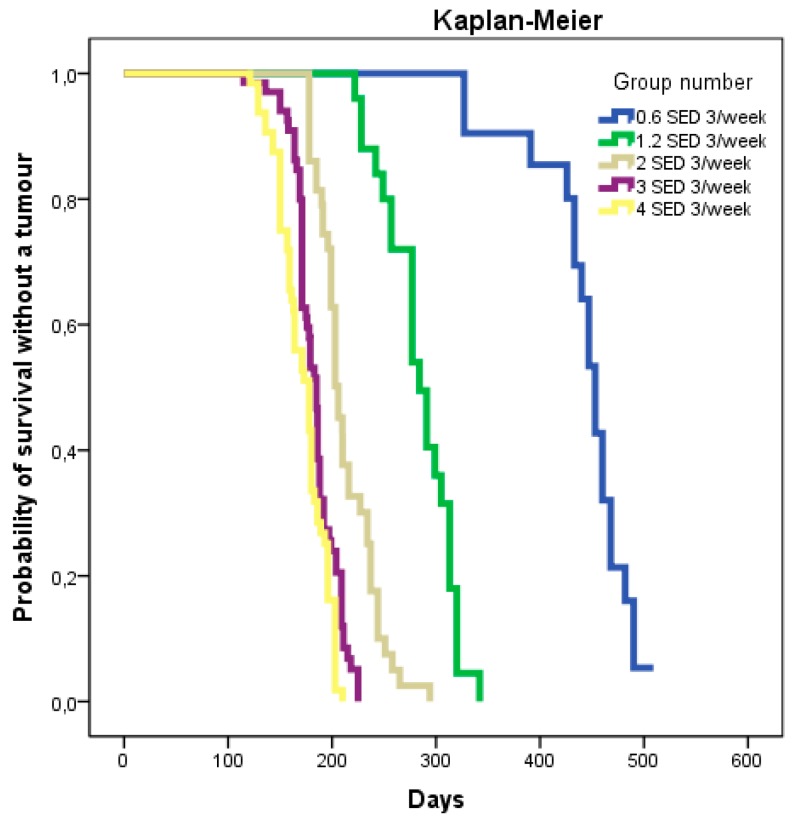
Dose–response study: A Kaplan–Meier plot showing tumor-free survival (Groups 6–10). The Kaplan–Meier plot demonstrates that there are significant differences in time until tumor development between the 5 groups irradiated with 0.6, 1.2, 2, 3 or 4 SED 3×/week, *p* < 0.005.

## Abbreviations

UVR	ultraviolet radiation
MED	minimal erythema dose
SCC	squamous cell carcinoma
SED	standard erythema doses
